# Influence of Scaffold Structure and Biomimetic Properties on Adipose Stem Cell Homing in Personalized Reconstructive Medicine

**DOI:** 10.3390/biomimetics10070438

**Published:** 2025-07-03

**Authors:** Doina Ramona Manu, Diana V. Portan, Monica Vuţă, Minodora Dobreanu

**Affiliations:** 1 Center for Advanced Medical and Pharmaceutical Research, “George Emil Palade” University of Medicine, Pharmacy, Science and Technology of Targu Mures, 38 Gheorghe Marinescu, 540142 Targu Mures, Romania; doina.manu@umfst.ro (D.R.M.); minodora.dobreanu@umfst.ro (M.D.); 2 Department of Mechanical Engineering and Aeronautics, University of Patras, Panepistimioupoli, 265 04 Patras, Greece; 3 Doctoral School of Medicine and Pharmacy, “George Emil Palade” University of Medicine, Pharmacy, Science and Technology of Targu Mures, 38 Gheorghe Marinescu, 540142 Targu Mures, Romania; monicavuta@yahoo.com; 4 Department of Laboratory Medicine, “George Emil Palade” University of Medicine, Pharmacy, Science and Technology of Targu Mures, 38 Gheorghe Marinescu, 540142 Targu Mures, Romania

**Keywords:** adipose stem cell, homing mechanism, regenerative therapies, biomaterials, scaffold engineering

## Abstract

Human adipose stem cells (ASCs) are multipotent cells expressing mesenchymal stem cell (MSC) markers that are capable of multilineage differentiation and secretion of bioactive factors. Their “homing” to injured tissues is mediated by chemokines, cytokines, adhesion molecules, and signaling pathways. Enhancing ASC homing is critical for improving regenerative therapies. Strategies include boosting chemotactic signaling, modulating immune responses to create a supportive environment, preconditioning ASCs with hypoxia or mechanical stimuli, co-culturing with supportive cells, applying surface modifications or genetic engineering, and using biomaterials to promote ASC recruitment, retention, and integration at injury sites. Scaffolds provide structural support and a biomimetic environment for ASC-based tissue regeneration. Natural scaffolds promote adhesion and differentiation but have mechanical limitations, while synthetic scaffolds offer tunable properties and controlled degradation. Functionalization with bioactive molecules improves the regenerative outcomes of different tissue types. Ceramic-based scaffolds, due to their strength and bioactivity, are ideal for bone healing. Composite scaffolds, combining polymers, ceramics, or metals, further optimize mechanical and biological properties, supporting personalized regenerative therapies. This review integrates concepts from cell biology, biomaterials science, and regenerative medicine to offer a comprehensive understanding of ASC homing and its impact on tissue engineering and clinical applications.

## 1. Introduction

Regenerative medicine is an interdisciplinary field that focuses on repairing, replacing, or regenerating aged, disease-affected, or damaged tissues and organs by leveraging the body’s natural healing mechanisms. It combines principles from stem cell biology, biomaterials, gene therapy, and tissue engineering to develop therapies that restore normal function in diseased or injured tissues.

Studies on biomaterials proved that cellular responses, including cell morphology, motility, proliferation, protein abundance, adhesion, and gene regulation, can be modulated by applying an efficient biomimetic design to biomaterials. Meanwhile, the potential range of stem cell applications in tissue engineering continues to grow in convergence with appropriate implants capable of creating tightly defined artificial microenvironments for each targeted organ [1]. Advancements in biomaterial manufacturing have enabled a shift from composites produced through complex concepts [2,3] to nano-processed materials, such as orthopedic metals [4,5], to multi-phase functional materials that incorporate metals, carbon-based elements, plastics [6], and sometimes 3D-printed components [7]. Moreover, autologous cell therapy using patients’ own cells may be delivered via 3D materials to deliver precise and ideal treatment through a personalized medicine approach [8].

Stem cell therapy uses MSCs within different tissues (bone marrow, adipose, placental, limb bud, amniotic fluid, dental, skin) or induced pluripotent stem cells to regenerate tissues through multiple mechanisms. One involves the direct replacement of damaged cells and tissues, while another relies on paracrine signaling, which modulates the microenvironment [9,10,11]. This includes activating the native immune response, exerting anti-inflammatory effects, preventing fibrosis, alleviating pain through cytokine secretion, regulating cell death, and providing immunomodulatory benefits [12,13,14]. Meanwhile, tissue engineering is developing bioengineered scaffolds [15] that support cell growth and tissue regeneration for applications such as skin grafts, cartilage or bone repair, and organ regeneration [16].

Adipose-derived stem cells (ASCs) have emerged as a promising tool in regenerative medicine due to their accessibility, multipotency, and immunomodulatory properties [17,18,19]. A critical factor in their therapeutic success is efficient homing, the process by which ASCs migrate and engraft at target tissues. Understanding the biological mechanisms governing ASC homing, as well as the role of scaffold materials in directing this process, is essential for optimizing regenerative strategies [20,21].

This review explores the key aspects of ASC homing, beginning with the biological mechanisms that drive their migration, including chemotaxis, receptor–ligand interactions, and major signaling pathways in contact with innovative biomaterials. Scaffolds reproduce the anatomy and physiology of tissue. An ideal scaffold should possess some attributes such as (1) porous structure, (2) biocompatibility, (3) bioactivity, and (4) excellent mechanical properties [22]. The validation of its biomimetic efficiency for fast biointegration involves primary cell cultures [23]. In this context, we examine the influence of scaffold chemical composition, comparing natural, synthetic, and hybrid biomaterials and their impact on ASC adhesion and migration.

We then discuss strategies for enhancing homing efficiency, such as chemotactic signaling to promote ASC migration to injury sites; immunomodulation that enhances ASC retention; and preconditioning strategies such as hypoxia, mechanical stimulation, and inflammatory cytokine exposure to further improve ASC adhesion and survival. Co-culturing with specific tissue cells enhances integration, while genetic engineering enhances migratory capacity and tissue targeting. Smart scaffolds with tunable properties provide an optimal microenvironment for ASC recruitment, viability, and differentiation. Functionalization with bioactive molecules improves targeted homing and adhesion, while advanced biomaterials, including 3D-printed, injectable, or electrospun scaffolds, support ASC retention and guided differentiation for tissue regeneration. By integrating insights from cell biology, biomaterials science, and regenerative medicine, this review aims to provide a comprehensive understanding of ASC homing and its implications for tissue engineering and clinical applications. Finally, we provide current challenges and future directions in scaffold-based ASC therapies.

## 2. Phenotype and Functional Properties of Human Adipose Stem Cells

ASCs are a rich, ubiquitous, and easily accessible source of multipotent stem cells [24]. Based on isolation methods from adipose tissue, ASCs, stromal vascular fraction (SVF), and micronized adipose tissue (MAT) can be obtained. The term ASCs specifically refers to mesenchymal stem cells (MSCs) extracted from adipose tissue and expanded in culture. SVF is obtained through a process involving centrifugation and enzymatic digestion with collagenase, which releases a heterogeneous cell population from the collagen matrix, such as ASCs, pericytes, smooth muscle cells, endothelial cells, immune cells, and progenitor cells. In contrast, MAT is produced through the mechanical separation of adipose tissue without collagenase, allowing cells to be released from the lipoaspirate with minimal manipulation. Unlike SVF, MAT retains the extracellular matrix (ECM) and ASCs, fibroblasts, and pericytes embedded within the ECM. Mechanical methods yield lower nucleated cells retained in niche, and lower frequencies of progenitor cells, than the enzymatic approaches. ASCs are localized in perivascular niches, and mechanical isolation methods release fewer cells from these spaces due to reduced ECM disruption compared with enzymatic methods [25,26].

Mechanical methods were initially developed to isolate “minimally manipulated” cells to potentially bypass strict FDA regulations, whereas enzymatic methods produce cells considered “more than minimally manipulated” and are regulated as drugs; however, recent FDA guidance aims to classify all SVF isolation methods, both enzymatic and mechanical, as yielding “more than minimally manipulated” cells, subjecting them to stricter oversight [27,28].

**Figure 1:**
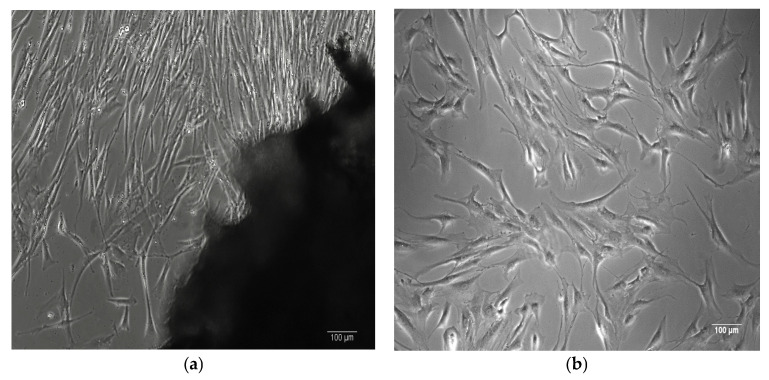

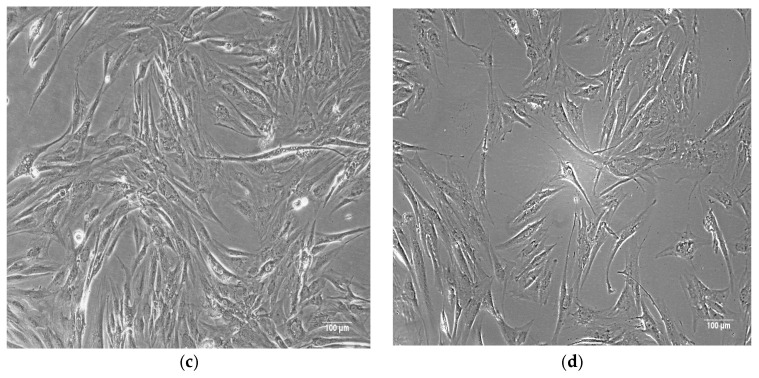
(**a**) Human bone-derived MSCs migrating from a bone explant. (**b**) Human bone-derived MSCs proliferating at passage 1. (**c**,**d**) Human ASCs proliferating at passage 1. Images captured at 100× magnification using phase-contrast microscopy.

The stem cells isolated from adipose tissue exhibit a characteristic spindle-shaped, bipolar morphology typical of mesenchymal stem cells (MSCs) [29], as shown in Figure 1.

Primary cells were isolated from human tissues by the authors at the Department of Immunology, Center for Advanced Medical and Pharmaceutical Research, Târgu Mureș.

**Figure 2:**
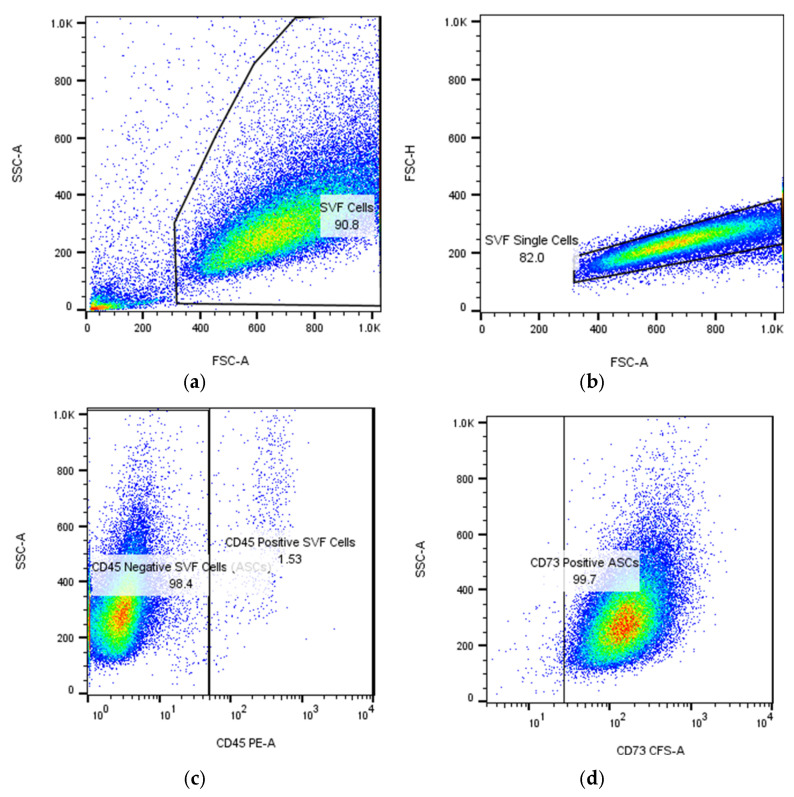

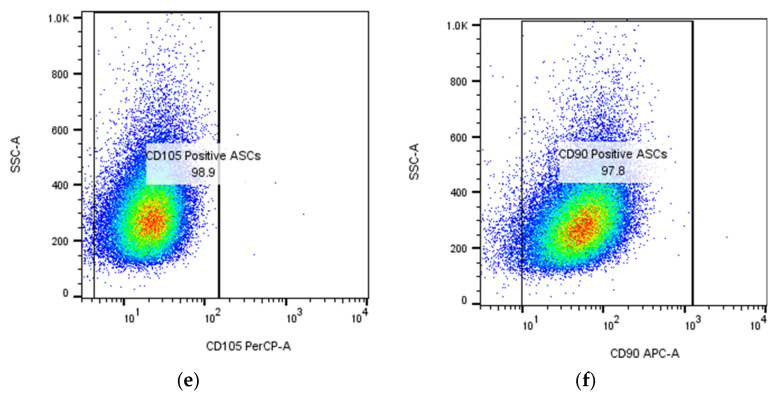
Flow cytometric identification in BD FACS Aria III of adipose-derived stem cells (ASCs) expanded in culture after passage 1 (P1): (**a**,**b**) gating strategy to isolate single cells; the numbers represent the percentage of cells and singlets, respectively; (**c**) detection of ASCs as CD45-negative cells (more than 98%); (**d**–**f**) expression of mesenchymal stem cell markers CD73, CD90, and CD105 on ASCs. The cells were isolated, cultured, submitted to passage 1, re-cultured until confluence, and then analyzed in flow cytometer at the Department of Immunology, Center for Advanced Medical and Pharmaceutical Research, Târgu Mureș.

ASCs express typical MSC markers, including receptor molecules (CD90 and CD105) and the GPI-anchored enzyme CD73 (CD105, CD73, and CD90 are expressed on virtually all mesenchymal stem cells, regardless of source), as illustrated in Figure 2.

ASCs also express adhesion molecules characteristic of different tissues (CD29 in placental MSCs; CD44 in adipose, skin, and dental MSCs; CD146 in endometrial MSCs; and CD166 in skin MSCs). Additionally, markers such as CD13, CD10, CD49e, and CD59 are usually positive [30].

In low-passage cultures, ASCs often express CD34, a marker commonly used to identify hematopoietic or endothelial progenitor cells, although its expression is influenced by passage number and culture conditions. Typically, ASCs show minimal expression of hematopoietic markers (CD11b, CD14, CD19, and CD45) and endothelial markers (CD31 and HLA-DR) [31].

ASCs are considered relatively immune-privileged because they lack HLA-DR (MHC class II) and costimulatory molecules (e.g., CD80, CD86, CD40, and CD40L) required for T- and B-cell activation. Moreover, they express low levels of MHC class I [19,32].

In early passages, ASCs express low levels of stemness-related transcriptional factors such as Oct4, Nanog, and Sox2, which are essential for pluripotency, self-renewal, proliferation, and survival of MSCs [33,34]. These pluripotency markers were barely detectable in a study conducted by Hatzmann et al. [35]. In another study, Pierantozzi et al. showed that Nanog, but not OCT-4 or SOX-2, is expressed in cultured MSCs, appearing only after in vitro culture and restricted to proliferating cells. Its expression declines in late passages for adipose and heart-derived MSCs. Despite this, Nanog levels do not correlate with proliferation, differentiation, or stemness, suggesting its activation reflects adaptation to in vitro conditions rather than a regulatory role [36].

The maintenance and regulation of pluripotency involves a complex interplay between intrinsic factors like Oct4, Sox2, and Nanog, and extrinsic signals such as the LIF/STAT3 pathway, acting in a context-dependent manner. Oct4 exerts dose-dependent effects on stem cell fate, promoting pluripotency at intermediate levels and driving differentiation at low or high levels through partner switching with Sox2 or Sox17. Its activity and stability are finely regulated by post-translational modifications such as phosphorylation, ubiquitination, and sumoylation, which influence its DNA binding, protein interactions, and functional roles in pluripotency and differentiation [37].

ASCs from both younger and older donors exhibit similar antigen profiles [38]. Single-cell RNA sequencing analysis has revealed low transcriptomic heterogeneity among ASCs [31,39,40,41,42,43]. However, no exclusive marker has yet been identified to distinguish ASCs; notably, ASCs do not express markers specific to bone marrow-derived MSCs, such as adhesion marker CD106 or the sialoglycoprotein podocalyxin (PODXL) [44].

ASCs from obese donors exhibit reduced expression of stemness markers compared with those from lean individuals, while promoting a pro-inflammatory phenotype. White adipose tissue exists as subcutaneous and visceral fat, with ASCs from both sharing viability and markers but differing in motility, secretion, and gene expression. Subcutaneous ASCs have greater adipogenic and osteogenic potential, while visceral ASCs proliferate slower and secrete more inflammatory cytokines. ASCs in superficial layers show angiogenic capacity, whereas deeper ASCs resemble visceral ASCs [45].

ASCs produce a wide range of soluble mediators, including cytokines, chemokines (both pro- and anti-inflammatory), adipokines, antioxidative molecules, pro-angiogenic and anti-apoptotic factors, and growth factors such as Vascular Endothelial Growth Factor (VEGF), Hepatocyte Growth Factor (HGF), Fibroblast Growth Factor (FGF), and Insulin-like Growth Factor (IGF-1). ASCs also secrete brain-derived neurotrophic factor (BDNF) and various interleukins (e.g., IL-1Ra, IL-6, IL-7, IL-8, and IL-11). Furthermore, extracellular vesicles (EVs) derived from ASCs transfer functional biomolecules (proteins; nucleic acids such as mRNA, microRNAs, tRNAs, and other non-coding RNAs; and lipids) that influence cell proliferation, migration, apoptosis, immune modulation, angiogenesis, metabolism, nerve regeneration, and tumorigenesis [19,46].

In addition to these phenotypic characteristics, ASCs possess multilineage differentiation potential, an essential property for their use in cell therapy and tissue engineering. They can differentiate into cells of ectodermal, endodermal, and mesodermal lineages. Notably, ASCs can give rise to adipogenic, osteogenic, chondrogenic, myogenic, angiogenic, cardiomyogenic, tenogenic, and periodontogenic cell types, reflecting their mesodermal origin. Their neuro-regenerative potential has also been evaluated [47]. In vitro differentiation is typically induced by culture in media supplemented with lineage-specific factors [24,48].

Under specific induction conditions, ASCs can transdifferentiate beyond mesodermal lineages (e.g., adipocytes, osteocytes) into ectodermal lineages (neurons, glial cells) [49,50], endodermal lineages (hepatocyte-like cells, pancreatic β-like cells) [51], or cardiomyocyte-like cells [52].

This plasticity can be driven by epigenetic remodeling, via chromatin modification or DNA methylation, by lineage-transcription factor overexpression or microRNA regulation [53,54].

ASCs transdifferentiation also occurs under niche signals such as specific growth factor/cytokine signaling (e.g., FGF, EGF, HGF) [55,56].

However, several limitations must be considered regarding this process. Stem cell transdifferentiation may be incomplete; although the resulting cells often express markers of the target lineage, they may not fully replicate their function. This requires stable reprogramming of the cell’s genome and epigenetic modifications. Functional integration into host tissue and long-term survival remain significant challenges.

The culture and expansion of ASCs, as well as freezing and long-term storage at ultra-low temperatures, are preparative steps that may influence their phenotype and function. Human ASCs retain their stemness in serum-free conditions, though FBS deprivation for 48 h reduces metabolism and proliferation without inducing apoptosis or necrosis. Alternative FBS substitutes such as human platelet lysates show superior support for hASC proliferation while maintaining their undifferentiated state. Similarly, thrombin-activated platelet-rich plasma (tPRP) and pooled human serum sustain ASC properties, though tPRP may reduce reactive oxygen species (ROS) accumulation and genetic instability, which can be mitigated by lowering the culture temperature [57].

Although cryopreserved lipoaspirate-derived cells show reduced viability and lower colony-forming unit percentages compared with fresh cells, the remaining viable cells retain adhesive and proliferative properties, which can offset the negative effects with continued growth. Prolonged cryopreservation at −70 °C further reduces both the number of viable cells and their viability [58].

ASCs rapidly undergo replicative senescence and lose stem cell properties after 21 days in 2D culture. Senescent cells exhibit decreased proliferation, morphological changes, and increased ROS, raising concerns about ASC stability and safety for application. Immortalization techniques, such as ectopic telomerase reverse transcriptase (TERT) expression, can prevent senescence and maintain proliferative and differentiation potential. While TERT overexpression has successfully immortalized ASCs in some studies, issues like chromosomal aberrations and loss of differentiation ability remain [59]. Co-transducing hTERT with other genes, like Bmi-1 or HPV-E6/E7, has also been explored, but results vary. Gene editing can immortalize cells, but karyotype variation must be considered [60,61].

## 3. ASC Homing

In stem cell science, homing refers to the ability of stem cells to locate their specific niches. This process involves preparing the niche, enhancing cellular migratory responsiveness, and increasing sensitivity to niche-derived signals. Stem cell homing mechanisms can be categorized as either non-systemic or systemic.

Non-systemic homing involves the recruitment of local or transplanted stem cells that navigate along a chemokine gradient released from an injured tissue site. Direct application of ASCs has proven effective for tissue regeneration in various conditions, including intracardiac, intra-articular, intramuscular, intraosseous, and intrathecal. MSC retention can be enhanced through targeted delivery methods such as intracerebral administration for neurological disorders, intratracheal delivery for lung disease, or intramyocardial injection for cardiac conditions [62].

Systemic homing occurs following intravascular transplantation. In this process, stem cells exhibit a migration pattern similar to that of leukocytes, enabling them to home to sites of injury or inflammation. This mechanism is mediated primarily by interactions among chemokines, cytokines, and adhesion molecules. For instance, in stromal cells derived from human adipose tissue, galectin-1 and CD24 have been identified as potential ligands for P-selectins [63,64]. ASCs express chemokine receptors such as CXC chemokine receptor (CXCR) type 4 and type 7, which respond to stromal cell-derived factor-1 (SDF-1/CXCL12). The CXCL5/CXCR2 axis induces ASC migration [65]. Additionally, monocyte chemoattractant protein-1 (MCP-1) enhances homing by binding to CC chemokine receptor type 2 (CCR2). The signaling molecules Talin and Kindlin interact with very late antigen-4 (VLA-4) upon SDF-1 activation, promoting migration. Moreover, the vascular cell adhesion molecule 1 (VCAM-1) expressed on endothelial cells binds to activated VLA-4 integrin. ASCs traverse endothelial cell junctions in response to inflammatory signals—a process known as transendothelial migration—and secrete matrix metalloproteinases (MMPs) to degrade extracellular matrix components, facilitating their infiltration into damaged tissues. Lysophosphatidic acid (LPA), a small bioactive phospholipid expressed in ASCs, stimulates ASC migration via the G protein-coupled receptor [66]. Mitogen-activated protein kinases (MAPKs), including Jun N-terminus kinase (JNK), p38, and extracellular signal-regulated kinase 1/2 (Erk1/2) are involved in stem cell migration. Erk1/2 induces mobility via the SDF-1/CXCR4 [67,68,69] and CXCL11/CXCR3 axis [70]. ASC migration and proliferation are enhanced via activation of the phosphatidylinositol-3 kinase (PI3K)/Akt signaling pathway [71]. ASC migration significantly increased through platelet-derived growth factor receptor-β (PDGFR)-β signaling, leading to oxygen species generation and microRNA-210 up-regulation [72]. Inflammatory chemokines also contribute to MSC migration [21].

A thorough understanding of the molecular events underlying ASC homing is essential for developing strategies to optimize this process for therapeutic applications.

## 4. Strategies to Enhance ASC Homing for Improved Regenerative Outcomes

**Figure 3:**
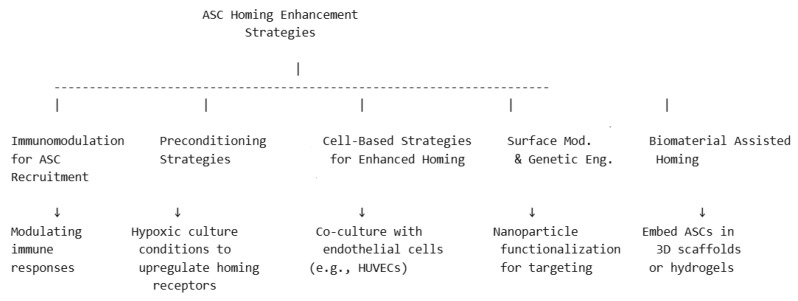
Schematic representation of strategies for ASC enhanced homing.

Enhancing ASC homing to target tissues is crucial for improving regenerative outcomes. Several strategies have been developed to optimize ASC recruitment, retention, and functional integration at injury or defect sites. The main mechanisms may be seen in the diagram in Figure 3, followed by detailed descriptions below.

### 4.1. Chemotactic Signaling

The SDF-1α/CXCR4 axis guides ASC migration to injury sites where SDF-1α is upregulated [73]. VEGF and angiopoietin-1 can enhance ASC recruitment by promoting vascularization, which in turn improves ASC delivery to damaged tissues [74]. PDGF and FGF stimulate ASC proliferation and migration via the activation of PI3K/Akt and MAPK pathways [75].

### 4.2. Immunomodulation for ASC Recruitment

Modulating immune responses with IL-4, IL-10, or TGF-β promotes an anti-inflammatory environment that, in turn, enhances ASC retention and differentiation. Reducing complement-mediated ASC clearance using complement inhibitors (e.g., C3a or C5a blockers) enhances ASC survival and engraftment. Promoting regulatory T cell (Treg) expansion reduces local inflammation, improving ASC homing and integration [13,76].

### 4.3. Preconditioning Strategies

Culturing ASCs under hypoxic conditions upregulates SDF-1 α and VEGF, enhancing migration and survival after transplantation [77].

Pre-exposing ASCs to fluid shear stress enhances their ability to adhere to vascular endothelial cells [78].

Exposure to inflammatory cytokines like TNF-α and IL-6 increases ASC expression of adhesion molecules (ICAM-1, VCAM-1), improving retention at injury sites [79,80]. Pre-treating ASCs with lipopolysaccharides (LPSs) has been shown to enhance wound healing and angiogenesis, while also increasing the secretion of growth factors involved in tissue regeneration and immune modulation [81].

Mechanical stretching or electrical stimulation can enhance ASC responsiveness to chemotactic cues, increasing homing efficiency [82,83].

Attaching magnetic nanoparticles to ASCs allows for targeted delivery using external magnetic fields, increasing homing efficiency [84].

### 4.4. Cell-Based Strategies for Enhanced Homing

Co-culturing ASCs with endothelial cells [85] or fibroblasts [86] can mimic the native microenvironment, preconditioning ASCs for better migration and integration upon transplantation [87].

For nerve regeneration, ASCs co-seeded with Schwann cells improve homing to injured peripheral nerves and enhance axonal growth [88].

Forming ASC spheroids with supporting cells (e.g., pericytes, fibroblasts) enhances their survival and homing efficiency [89].

### 4.5. Surface Modification and Genetic Engineering

Functionalizing ASCs with nanoparticles loaded with SDF-1α, peptides, or growth factors improves targeted homing, adhesion, proliferation, and stem cell differentiation [90]. Modifying ASC membranes with homing peptides increases their recruitment to injured sites [91].

Engineering ASCs to overexpress CXCR4 or VEGF enhances their migratory capacity and adhesion to endothelial cells [87,92,93,94].

### 4.6. Biomaterial-Assisted Homing

**Figure 4:**
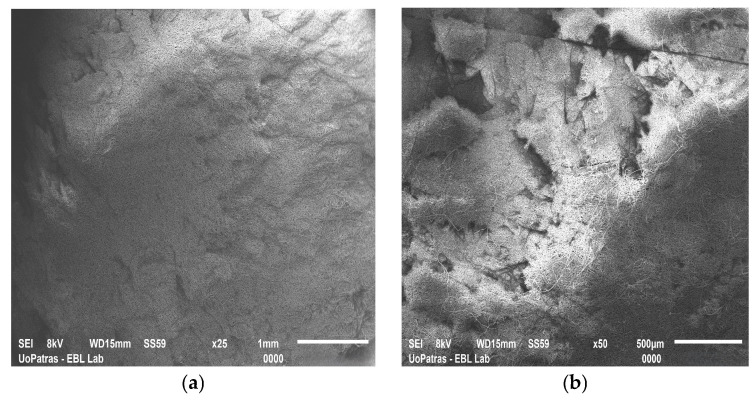

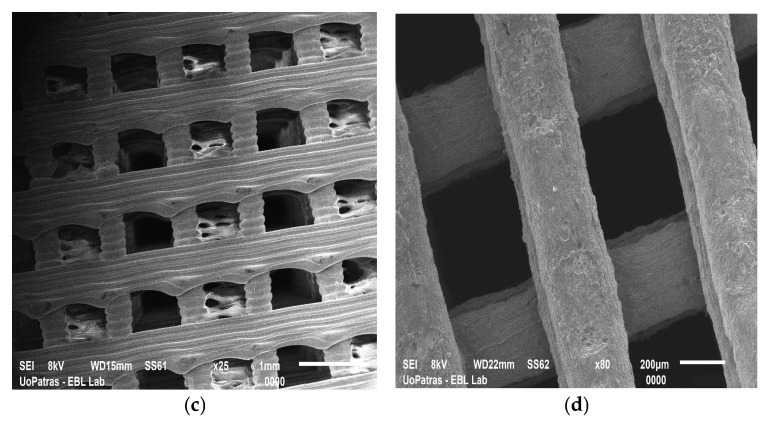
SEM images of (**a**) graphene nanoplatelet-reinforced polyetherimide ketone scaffold fabricated by electrospinning; (**b**) double reinforcement: graphene nanoplatelet- and hydroxyapatite-reinforced polyetherimide ketone scaffold fabricated by electrospinning; (**c**) polylactic acid scaffold fabricated by 3D printing; and (**d**) carbon nanotube-reinforced polylactic acid scaffold fabricated by 3D printing. The images have been provided by the Department of Mechanical Engineering and Aeronautics from the University of Patras.

Current studies suggest that incorporating ASCs within 3D scaffolds may offer a promising alternative for wound healing, orthopedic tissue repair, cardiovascular grafts, and post-surgical tissue reconstruction. The ideal scaffolds create a biomimetic environment that supports ASCs’ viability, proliferation, and differentiation. Furthermore, scaffolds designed to encapsulate ASCs can stimulate their differentiation into specific cell types. Advanced fabrication techniques allow tailoring scaffolds’ features and functionality. Between them, electrospinning and 3D printing are the most popular; scaffolds manufactured by the authors may be seen in Figure 4.

**Figure 5:**
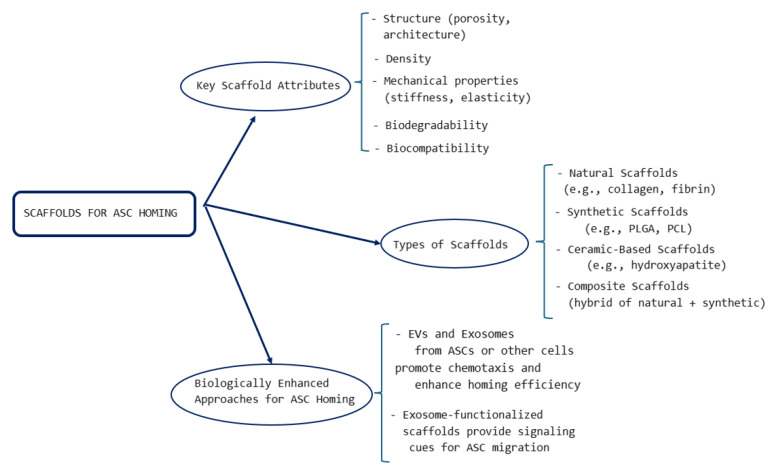
Schematic representation of the important aspects related to ASC homing via scaffolds.

In Figure 5, we may see a schematic representation of the most important aspects related to ASC homing via scaffolds. The decisive factors of successful homing are correlated with the scaffold features like structure, mechanical properties, biocompatibility, and others. There are several classes of scaffolds (natural, synthetic thermoplastic, ceramic-based scaffolds), while the procedures may be considerably enhanced by the addition of biological components. The more detailed descriptions are given in the sections below.

#### 4.6.1. Key Attributes of Scaffolds Used for Tissue Regeneration

A scaffold is a temporary or permanent template that provides structural support and an optimal environment for seeding stem cells during tissue regeneration. Scaffolds should be designed to mimic the regulatory functions of the extracellular matrix (ECM) in tissue formation with adjustable chemical, electrical, and physical properties [95].

Scaffolds can be porous, offering high surface area and enhanced cell migration. The architecture of porous scaffolds—including pore size and interconnectivity—must ensure proper cell attachment, cell-to-cell interactions, and migration. Porosity also facilitates tissue penetration, vascularization, and nutrient/oxygen diffusion, which are essential for cell survival [96,97]. Furthermore, pore size and geometry influence differentiation. Larger pores favor osteogenic differentiation, while smaller pores promote adipogenic differentiation [98].

Fibrous scaffolds composed of biocompatible polymers arranged in various scales, dimensions, and spatial configurations mimic the structural complexity of the ECM [99].

Another type of scaffold is hydrogels, which are highly hydrated networks formed by crosslinked polymer chains used for soft-tissue applications [100].

Derived from native tissues, decellularized ECM (dECM) preserves the architecture and mechanics of the ECM, is non-immunogenic, and offers advantages such as promoting tissue regeneration via chemotactic stimuli and the presence of native growth factors [101].

A scaffold must be biocompatible to promote cell adhesion, proliferation, migration, and ECM restoration. Scaffold bioactivity refers to its ability to enhance tissue regeneration via cell attachment ligands, growth factors that induce differentiation, and topographical cues for proper spatial organization. The biodegradation rate should be commensurate with tissue regeneration, and degradation products must be non-toxic and either incorporated into other biochemical pathways or eliminated without harming adjacent tissues [102].

Moreover, scaffolds should possess mechanical properties similar to the target tissue to support cell growth and mimic the native ECM. The elasticity and degradation rate should be tailored to match the target tissue, ensuring gradual replacement by the native ECM. Key mechanical parameters include the elastic modulus (also called Young’s modulus) for resistance to deformation under stress; tensile strength, providing maximum stress before failure under tension; fracture toughness for resistance to crack propagation; fatigue resistance, the ability to withstand cyclic loading over time; and elongation at break, providing stretchability before failure [103,104].

Additionally, scaffold stiffness influences lineage commitment—soft substrates favor adipogenesis and chondrogenesis, while stiffer substrates promote osteogenesis [105].

Common fabrication techniques include salt leaching, solvent casting, gas foaming, thermally induced phase separation, freeze-drying, electrospinning, thermally induced self-agglomeration, and three-dimensional (3D) printing [105]. These methods yield scaffolds with the mechanical and bioactive properties required for specific target tissues. Combining scaffold types can further enhance these properties and modulate degradation rates via microstructural adjustments. Incorporating bioactive factors into hydrogels, nanofiber scaffolds, microparticles, or bioprinted scaffolds ensures sustained, localized release that optimizes stem cell function and patient-specific tissue regeneration [102]. In particular, 3D bioprinting and electrospinning offer personalized scaffold architectures with effective bioactive factor delivery [106], while freeze-drying and hydrogel crosslinking remain valuable for tuning pore size and morphology [107].

#### 4.6.2. Natural Scaffolds for Adipose Stem Cell-Based Tissue Engineering

Natural scaffolds—typically derived from polysaccharides or proteins—mimic the extracellular matrix (ECM), providing a supportive microenvironment that promotes cell adhesion, proliferation, and differentiation. Natural scaffolds provide both physical and biochemical platforms that facilitate ASC attachment and growth, which are critical for tissue regeneration.

Hyaluronic acid (HA), a skin/connective tissue-derived glycosaminoglycan, aids wound healing by supporting fibroblast proliferation and keratinocyte migration; however, its low mechanical strength limits its use in high-strength applications [108]. Alginates, derived from algae and bacteria and forming gels via ion exchange when in contact with body exudates, are also used in wound healing [109].

Chitosan, a cationic polysaccharide, is valued for its antibacterial, hemostatic, and biodegradable properties, although its low mechanical strength restricts its high-load applications [110]. Carboxymethyl-chitosan, with improved strength, biocompatibility, and antibacterial activity, supports tissue regeneration and wound healing [111].

Bacterial cellulose (BC) is ideal for tissue engineering due to its purity, high porosity, and excellent biocompatibility; it aids wound care by maintaining a moist environment, absorbing exudates, and preventing infection. BC-based nanocomposites and hydrogels show promise for bone and cartilage regeneration [112].

Collagen, a major ECM protein, supports wound healing but has low load-bearing capacity, whereas gelatin (a collagen derivative) supports cell adhesion and can serve as a carrier for growth factors or drugs [113]. Constructs comprising human platelet-poor plasma, alginate, and fibrin gel exhibited enhanced adipogenic differentiation and VEGF expression. Although collagen sponges offered favorable mechanical properties and supported cell viability, they resulted in poor adipogenic differentiation. Conversely, fibrin gel was identified as the optimal compromise, providing superior mechanical properties, high cell viability, and stimulation of adipogenic and angiogenic factor secretion [114]. The porous structures (e.g., those made from collagen, HA, or BC), ensure adequate nutrient and oxygen supply, ASC migration, and ECM deposition. For soft tissue regeneration common naturally-derived scaffolds include collagen-HA, fibrin-HA [115,116], and gelatin-dECM [117]. These can be fabricated via electrospinning (producing nanofibrous ECM-mimicking structures) or freeze-drying (yielding highly porous constructs for cell infiltration) [118].

Hydrogels, formed through UV- or enzyme-mediated crosslinking, offer injectable or 3D matrices with tunable degradation [119]. For cartilage repair, 3D bioprinting enables precise hydrogel structuring to create patient-specific scaffolds [120], while freeze-drying combined with crosslinking improves the hydrogel’s mechanical properties and water retention for osteochondral defect repair [121]. Additionally, microfluidic gelation facilitates microsphere or microgel obtaining for cell encapsulation [122].

The primary objectives in cardiovascular tissue engineering are vascularization, endothelialization, and functional remodeling. Cardiovascular tissue engineering requires scaffold materials such as fibrin, collagen, and alginate-HA-dECM composites to support vascularization and remodeling [123,124]. ASCs can grow and proliferate well on Atelocollagen (a biological collagen from cattle), and induced by 5-azacytidine, the cells can differentiate into myocardial cells [125]. Techniques like electrospinning with cell seeding produce fibrous scaffolds that mimic blood vessel walls [126], while 3D printing of hydrogels enables the formation of vascularized networks that support endothelial cell growth and stem cell differentiation [127,128].

For nerve regeneration, scaffolds composed of collagen–laminin, chitosan–alginate, and fibrin–HA–dECM facilitate axonal growth and functional recovery. Electrospinning can create aligned nanofibers that serve as nerve guidance conduits [84,129], while 3D bioprinting with cell-laden hydrogels enables precise nerve conduit designs that enhance neural differentiation [130]. Freeze-drying of collagen-hydrogel models and scaffolds with laminin coating increase scaffold bioactivity for spinal cord and brain tissue engineering [131]. Nerve tissue engineering focuses on neurogenic differentiation, axonal outgrowth, and functional recovery. A co-culture system of Schwann cells and ASCs seeded into a silk fibroin/collagen scaffold was able to construct a tissue-engineered nerve conduit [132,133].

ASCs possess an intrinsic myogenic potential and can differentiate into skeletal myoblasts, as indicated by the expression of markers such as MyoD, Myogenin, and α-skeletal actinin. In one study, a cross-linked HA scaffold loaded with ASCs positive for NG2, a pericytic surface marker, differentiated into skeletal muscle tissue [134].

The naturally derived scaffolds can be functionalized with growth factors, cytokines, or other bioactive molecules to enhance ASC differentiation into specific cell types (e.g., adipocytes, osteocytes, chondrocytes) based on therapeutic needs. These scaffolds can be enriched with bioactive factors such as VEGF, angiopoietin-2 (Ang-2), elastin, basic fibroblast growth factor (bFGF), or transforming growth factor-β1 (TGF-β1), to stimulate blood vessel formation, fibroblast proliferation, ECM production, and adipogenesis, while HA fragments and PDGF further enhance hydration, cell migration, and tissue repair [135,136,137]. Hydrogels infused with SDF-1α or HA promote localized ASC retention and survival post-injection [138]. ECM-mimicking scaffolds incorporating growth factors improve ASC adhesion and differentiation at the target site [139]. In the particular case of optimal cartilage regeneration, scaffolds should promote chondrogenic differentiation, ECM accumulation, and mechanical strength. The incorporation of bioactive factors such as bone morphogenetic protein-2 (BMP-2), BMP-7, and IGF-1 enhances chondrocyte proliferation and ECM formation and supports cell survival [140,141], while chondroitin sulfate and heparan sulfate improve chondrocyte adhesion [142]. Advanced microparticle-loaded hydrogels providing sustained release of TGF-β1, SDF-1, BMP-2, and IGF-1, as well as chitosan–collagen 3D scaffolds infused with growth factors, have been developed for long-term cartilage repair [143,144]. In cardiovascular disease, key bioactive factors for vascular repair include VEGF (to promote vessel formation), angiopoietin-1 (to stabilize new vessels) [134], and SDF-1α (to recruit endothelial and smooth muscle progenitors [145,146]. Nitric oxide-releasing compounds improve endothelial function and reduce thrombosis, while bFGF and fibronectin enhance cardiomyocyte proliferation. Scaffolds such as injectable HA–fibrin hydrogels loaded with VEGF and SDF-1α [136] and electrospun vascular grafts coated with nitric oxide-releasing polymers further optimize regeneration [147].

In neurological disorders, bioactive factors such as nerve growth factor (NGF) promote stem cell differentiation into neural-like cells, while brain-derived neurotrophic factor (BDNF) and glial cell-derived neurotrophic factor (GDNF) enhance neurite outgrowth and Schwann cell function [148]. Additionally, chondroitinase ABC degrades inhibitory ECM molecules to facilitate nerve regeneration [145], and laminin plus fibronectin improve cell adhesion [149]. Aligned electrospun nanofibers coated with NGF and BDNF serve as effective nerve conduits, and hydrogel-based drug delivery systems provide the controlled release of neurotrophic factors [150].

Controlled release of homing factors from biodegradable scaffolds enhances ASC recruitment and integration [144]. Being biodegradable, they allow gradual replacement by new tissue, reducing the risk of long-term inflammation or rejection, which is particularly advantageous in clinical applications.

Platelet-rich plasma (PRP), derived from the patient’s blood, enhances tissue healing and regeneration by promoting cell adhesion, angiogenesis, and tissue repair [151]. Ghiasi et al. demonstrated that ASC viability and proliferation were significantly higher in active platelet-rich plasma compared with fibrinogen glue and alginate, likely due to the abundant release of growth factors and cytokines [152].

dECM derived from native tissues preserves the structural and biochemical properties needed to support tissue regeneration while minimizing immune rejection [153,154]. In a study by Flynn et al. [155], decellularized human placenta facilitated ASC attachment and spreading, while adipogenic differentiation was enhanced when cells were encapsulated in non-cell-adhesive, crosslinked hyaluronan scaffold gels. In contrast, lower cell viability was observed when ASCs were encapsulated versus when they adhered to the scaffold matrix; moreover, non-adhesive scaffolds promoted adipogenic differentiation relative to cell-adhesive matrices. In 2022, Ren et al. [46] published findings showing that seeding ASCs in decellularized adipose tissue ECM facilitated wound healing by enhancing proliferation, angiogenesis, epithelization, and anti-inflammatory effects. The decellularized adipose tissue ECM preserved its 3D structure and key components—including collagen, sulfated glycosaminoglycans, and VEGF—while lacking MHC class I. Cartilage dECM-derived scaffolds with a 3D interconnected porous structure, when seeded with ASCs, have shown cartilage tissue formation [156]. Acellular cartilage matrices, which have excellent physicochemical properties and biocompatibility, are ideal for cartilage tissue engineering [157]. dECM processing combined with bioreactor culturing preserves native ECM signals for use in heart patches and vascularized constructs [158]. Liver dECM preserves the macroscopic 3D architecture, native composition, and ultrastructure and has proven valuable for promoting ASCs’ differentiation into hepatocytes in both the presence and absence of growth factors [159].

These insights underscore the importance of scaffold selection and functionalization in targeted tissue engineering applications. Although naturally derived scaffolds offer benefits such as biocompatibility, biodegradability, bioactivity, and reduced immune response, they also present some limitations (e.g., low mechanical strength, rapid degradation, limited tunability). The main types and characteristics of naturally derived scaffolds are summarized in Table 1. The challenges can be overcome by combining natural scaffolds with bioengineering techniques to enhance their structural and functional properties for more effective ASC-based regenerative medicine.

#### 4.6.3. Synthetic Scaffolds for Tissue Regeneration

Natural polymers offer excellent biocompatibility and cell adhesion, while synthetic polymers allow better control over porosity and provide controlled degradation, tunable physical, chemical, and mechanical properties, and scalability for mass production. Their tissue integration depends on optimizing degradation rates, mechanical properties, and bioactivity [160,161].

Synthetic scaffolds can be categorized into biodegradable and non-biodegradable types. The main synthetic scaffolds with their characteristics are summarized in Table 2.

Biodegradable synthetic scaffolds gradually degrade, allowing natural tissue to replace them. Common materials include polylactic acid (PLA), which degrades slowly and is used in bone and cartilage regeneration [162,163]. However, resorbable polymers like PLA have limited osteoinductive capacity [164]. Polyglycolic acid (PGA) degrades more rapidly and is used in sutures and soft tissue repair; poly (lactic-co-glycolic acid) (PLGA) is a copolymer with adjustable degradation properties used in drug delivery and scaffolding [165]; polycaprolactone (PCL) has a longer degradation time and high mechanical strength, and is suitable for bone and nerve regeneration [166,167]. Nanofibrous scaffolds such as electrospun PCL or PLGA mimic the ECM, improving cell attachment and differentiation [168].

**Table 2:** Summarizing synthetic scaffolds used in studies illustrating advancements in strategies for enhancing ASC homing and differentiation.

Synthetic Scaffolds	Material	Properties	Applications	References
Polylactic acid (PLA)	Biodegradable polymer	Slow degradation, low osteoinductive capacity	Bone, cartilage regeneration	[162,163,164]
Polyglycolic acid (PGA)	Biodegradable polymer	Rapid degradation	Sutures, soft tissue repair	[165]
Poly (lactic-co-glycolic acid) (PLGA)	Copolymer	Tunable degradation, drug-delivery applications	Drug delivery, scaffolding	[165,167]
Polycaprolactone (PCL)	Biodegradable polymer	Long degradation time, high mechanical strength	Bone, nerve regeneration	[166,167,168]
Polyethylene glycol (PEG)	Non-biodegradable polymer	Biocompatible, hydrophilic	Drug delivery, cell encapsulation	[169,170]
Polymethylmethacrylate (PMMA)	Non-biodegradable polymer	High stability, used in bone cements	Orthopedics, dental applications	[171]
Polyurethane (PU)	Synthetic polymer	Flexible, high mechanical strength	Cardiovascular, orthopedic applications	[172]

Non-biodegradable synthetic scaffolds are used when long-term structural support is required. These materials exhibit strong mechanical properties but may require permanent implantation or later removal. Examples include polyethylene glycol (PEG), which forms hydrogels commonly used for drug delivery [169] and cell encapsulation [170]. PEG is widely used in synthetic scaffolds due to its biocompatibility, hydrophilicity, and low immunogenicity, supporting chondrocyte viability and ECM deposition. PCL, an FDA-approved polymer, offers tunable mechanical strength and can be fabricated as electrospun nanofibers or porous scaffolds. Polymethylmethacrylate (PMMA) offers stability in bone cements and dental applications [171]. Polyurethane (PU), valued for its flexibility and strength, is frequently used in cardiovascular and orthopedic applications, drug delivery, and wound healing [172]. In addition, PEG, PU, and PCL hydrogels are beneficial for skin and wound healing [173].

Because synthetic scaffolds inherently lack bioactivity, surface modifications with bioactive molecules (e.g., ECM proteins, RGD peptides, or growth factors) are often necessary to enhance cell adhesion, proliferation, and differentiation [174]. Incorporating VEGF, TGF-β, FGF, or BMPs further promotes stem cells’ differentiation and tissue regeneration [175]. Moreover, integrating controlled drug delivery [176] or gene delivery systems [10] within synthetic scaffolds can improve stem cell-based regenerative outcomes.

Integrating conductive polymers (e.g., polypyrrole, graphene) into scaffolds enhances ASC migration via electrical stimulation, which is beneficial for nerve and cardiac repair [129].

#### 4.6.4. Ceramic-Based Scaffolds

Ceramic-based scaffolds possess a 3D structure that facilitates cell attachment, proliferation, and differentiation, making them ideal for bone regeneration. In addition to biocompatibility, hydrophilicity, and bioactivity, these scaffolds must have porosity that supports nutrient diffusion and cell migration as well as mechanical strength comparable to native bone. While higher porosity may compromise the mechanical performance, lower porosity can hinder nutrient/oxygen diffusion and integration with the host bone. Ceramic materials offer mechanical strength similar to native bone and exhibit degradability that matches the rate of bone formation [177].

Bioactive ceramics used in scaffold fabrication include calcium phosphate-based materials such as hydroxyapatite, tricalcium phosphate (TCP), and biphasic calcium phosphate (BCP), a combination of hydroxyapatite and TCP. Electrical properties are important to ensure a biomimetic environment for the cell culture [178]. In this sense, hydroxyapatite offers excellent osteoconductivity, but it degrades slowly, whereas TCP is more favorable for bone remodeling [179]. TCP and hydroxyapatite promote osteoinduction but are brittle and poorly absorbed [164].

Bioactive glasses composed of silica-based materials form a bone-like apatite layer upon interacting with body fluids, releasing ions that enhance bioactivity. Low-alkali or nearly alkali-free bioactive glasses are particularly suitable for bone regeneration and tissue engineering [180].

Zirconia, silicon nitride, and alumina are bioinert ceramic-based scaffolds that provide high mechanical strength and antibacterial properties but exhibit lower bioactivity compared with calcium-based ceramics [181].

The fabrication of ceramic scaffolds can be optimized to achieve ideal architecture, porosity, and mechanical properties with the incorporation of bioactive ions or molecules to create patient-specific implants [182].

Ceramic-based scaffolds can serve as orthopedic and dental implants that promote bone healing and regeneration [183] and are used in craniofacial reconstruction to repair bone defects [184]. Ceramic scaffolds can also function as drug delivery systems, releasing growth factors or antibiotics in a controlled manner to enhance healing. Limitations in mechanical strength and bioactivity can be mitigated by incorporating nanoparticles or growth factors [185].

#### 4.6.5. Composite Scaffolds

Composite scaffolds provide tailored properties for personalized medicine and regenerative therapies by overcoming the limitations of single-component scaffolds. They enhance mechanical strength, bioactivity, and degradation control.

The most common composite scaffolds are polymer–ceramic composites, which combine the versatility and degradability of polymers with the bioactivity and mechanical strength of ceramics. The ceramic component enhances osteoconductivity and supplies the mineral fraction essential for bone regeneration. Such composites are widely used in orthopedic applications, bone grafts, and dental implants because they closely mimic the composition and structure of native bone [186,187,188]. Bone regeneration often relies on scaffold combinations such as collagen–hydroxyapatite–chitosan [189] or bone dECM with fibrin [190] to mimic the natural bone environment. In a study performed by Calabrese et al. [191], a composite material comprising mineralized type I collagen and magnesium-enriched hydroxyapatite, designed to mimic bone, was used to study osteogenesis and vasculogenesis during bone tissue regeneration. Collagen stimulates MSC differentiation into osteoblasts, and the incorporation of hydroxyapatite enhances cell proliferation, differentiation, and bone matrix formation. The collagen/Mg-HA scaffold recruited host cells in vivo that migrated into the scaffold, underwent osteogenic differentiation, and promoted vasculogenesis. Moreover, seeding human ASCs onto these scaffolds prior to implantation significantly improved bone regeneration. Techniques such as electrospinning combined with freeze-drying produce porous nanofibers that promote osteogenic differentiation [192], while sol-gel calcium phosphate-based materials enhance mineralization and accelerate stem cell differentiation [193]. Moreover, 3D bioprinting enables the creation of customized scaffolds for large bone defects that integrate bioactive molecules and growth factors [106].

Key objectives in bone regeneration include osteogenic differentiation, mineralization, and vascularization. Factors such as BMP-2 BMP-7, and BMP-9 stimulate osteoblast differentiation and bone matrix formation [139], while PRP growth factors enhance MSC osteogenic potential [194,195]. Controlled dexamethasone dosing further promotes osteogenesis, and calcium phosphate nanoparticles provide bioactive mineralization cues [196]. Improved scaffold integration may be achieved using bioceramic-coated scaffolds (e.g., with hydroxyapatite or tricalcium phosphate) or electrospun collagen scaffolds designed for sustained dexamethasone release [197]. In a study by Gao et al. [198], ASC-derived endothelial cells, alone or in co-culture with ASC-derived osteoblasts, were seeded onto a PLGA-based electrospun nanocomposite scaffold containing amorphous calcium phosphate (CaP) nanoparticles. The addition of CaP nanoparticles slowed cell proliferation but enhanced the differentiation of osteoblasts compared with pure PLGA scaffolds. Co-cultures of endothelial cells and osteoblasts promoted mutual differentiation. The CaP nanoparticles also improved the scaffold’s mechanical stability, bioactivity, and osteoconductivity, making it ideal for seeding with pre-differentiated cells.

Another category is polymer–polymer composites, which combine two or more polymers to optimize mechanical strength, degradation rate, and bioactivity. Natural blends (e.g., collagen with chitosan), enhance cellular interactions while remaining biodegradable, making them ideal for soft-tissue engineering. Synthetic combinations (e.g., PLGA with PCL) offer improved structural integrity and prolonged degradation for load-bearing applications. These composites are frequently used in cartilage repair, nerve regeneration, and wound healing. In addition, various bioactive materials have been employed to create injectable hydrogel scaffolds for cartilage regeneration [187,199].

Ceramic–metal composites combine the bioactivity of ceramics with the high mechanical strength of metals and are useful for load-bearing applications such as orthopedic and dental implants.

Metals like titanium (Ti), magnesium (Mg), and stainless steel enhance scaffold strength, addressing ceramic fragility. Metallic biomaterials may be bioinert (e.g., Ti and stainless-steel implants) or prone to corrosion (e.g., Mg- or Zn-based implants). Incorporating bioactive ceramics and polymers can improve surface bioactivity and corrosion resistance [200]. Magnesium-based composites are attractive for their biodegradability and bone-forming ability, whereas titanium-based composites offer excellent biocompatibility and corrosion resistance. These materials are common in spinal fusion devices, joint replacements, and maxillofacial reconstructions [201].

Hybrid composites incorporating nanomaterials have also gained attention due to enhanced mechanical and biological properties. Embedding nanoparticles such as carbon nanotubes (CNTs), silver nanoparticles, or silica nanoparticles improves scaffold strength, imparts antibacterial properties, and enables the delivery of bioactive molecules. For instance, silver nanoparticles reduce infection risks [202], while carbon nanotube-reinforced scaffolds provide electrical conductivity beneficial for neural tissue engineering and bone healing [203]. Such nanocomposites are promising smart scaffolds with controlled drug release and tissue-specific regeneration [159]. Ceramic and composite scaffold types, with their main characteristics, are summarized in Table 3.

### 4.7. Extracellular Vesicles (EVs) and Exosome-Based Approaches

Pre-treating ASCs with EVs or exosomes from injured tissues enhances their homing ability by activating migration-related pathways [204].

Loading ASCs with exosomes rich in SDF-1α, miR-21, HIF-1α, or other factors improves their ability to localize to ischemic and inflamed tissues [205]. Incorporating exosomes into hydrogels or scaffolds creates a sustained-release system that recruits ASCs to the injury site [206].

## 5. Conclusions

ASCs hold great promise in regenerative medicine due to their accessibility, multipotency, and immunomodulatory properties. Their ability to differentiate into multiple lineages and secrete bioactive factors makes them valuable for tissue repair. However, challenges such as donor variability, culture-induced changes, and regulatory constraints must be addressed. Optimizing ASC homing, including by using scaffolds designed to mimic the regulatory functions of the natural extracellular matrix and engraftment through better delivery strategies and molecular modulation, will enhance their therapeutic potential. Overcoming these hurdles is key to translating ASCs into effective, standardized clinical treatments.

## Figures and Tables

**Table 1:** Summarizing types of natural scaffolds used in studies, illustrating advancements in biomaterial-assisted strategies for enhancing ASC homing and differentiation.

Natural Scaffolds	Material	Properties	Applications	References
Hyaluronic acid (HA)	Glycosaminoglycan	Supports fibroblast proliferation, keratinocyte migration; low mechanical strength	Wound healing, soft tissue regeneration	[108]
Alginate	Polysaccharide (algae/bacteria)	Forms gels via ion exchange; good biocompatibility	Wound healing, cardiovascular applications	[109]
Chitosan	Cationic polysaccharide	Antibacterial, hemostatic, biodegradable; low mechanical strength	Tissue regeneration, wound healing	[110]
Carboxymethyl chitosan	Modified chitosan	Enhanced strength, biocompatibility, antibacterial activity	Wound healing, tissue regeneration	[111]
Bacterial cellulose (BC)	Microbial-derived polysaccharide	High porosity, excellent biocompatibility	Wound care, bone/cartilage regeneration	[112]
Collagen	ECM protein	Supports wound healing; low load-bearing capacity	Soft-tissue engineering, nerve regeneration	[113]
Fibrin	Protein-based hydrogel	Supports vascularization, cell viability	Cardiovascular, Soft-tissue engineering	[114]
Hydrogels	Collagen derivative	Supports cell adhesion; drug/growth factor carrier	Soft-tissue engineering	[102,119,120,121,122]
Platelet-rich plasma (PRP)	Blood-derived	Promotes angiogenesis, cell adhesion	Soft tissue repair, wound healing	[151,152]
Decellularized ECM (dECM)	Tissue-derived ECM	Maintains native ECM composition, biocompatible	Adipose, cartilage, liver, cardiovascular tissue engineering	[101,153,154,155,156,157,158,159]

**Table 3:** Summary of hybrid and composite scaffolds used in the study of ASC homing and differentiation.

Hybrid/Composite Scaffolds	Material	Properties	Applications	References
Collagen–HA	Protein–polysaccharide composite	Supports ASC migration, ECM deposition	Soft-tissue engineering	[189,191]
Fibrin–HA	Protein–polysaccharide composite	High biocompatibility, vascularization	Soft tissue, cardiovascular applications	[116]
Gelatin–dECM	Protein–tissue composite	Supports cell adhesion, tunable degradation	Soft-tissue engineering	[117]
Polymer–ceramic composites	PLGA, PCL, hydroxyapatite, tricalcium phosphate	Bioactive, osteoconductive	Bone regeneration, orthopedic implants	[186,187,188]
Ceramic–metal composites	Hydroxyapatite–titanium, magnesium	High mechanical strength, osteoinductive	Bone repair, orthopedic applications	[201]
Nanocomposite scaffolds	Electrospun PCL, PLGA, hydroxyapatite	ECM-mimicking, improves osteogenic differentiation	Bone, cartilage regeneration	[159,196,197,202,203]
Conductive polymers	Polypyrrole, graphene	Enhances ASC migration via electrical stimulation	Nerve, cardiac repair	[131]

## Abbreviations

The following abbreviations are used in this manuscript:

ANG-2	angiopoietin-2
ASC	adipose stem cell
bFGF	basic fibroblast growth factor
BC	bacterial cellulose
BCP	biphasic calcium phosphate
BDNF	brain-derived neurotrophic factor
BMP	bone morphogenetic protein
2D	bi-dimensional
CaP	calcium phosphate
CCR	C-C motif chemokine receptor
CXCL	C-X-C motif chemokine ligand
CXCR	C-X-C motif chemokine receptor
CNT	carbon nanotube
dECM	decellularized extracellular matrix
Erk1/2	extracellular signal-regulated kinase 1/2
ECM	extracellular matrix
EV	extracellular vesicle
FDA	Food and Drug Administration
FBS	fetal bovine serum
FGF	fibroblast growth factor
GDNF	glial cell-derived neurotrophic factor
GNP	graphene nanoplatelet
GPI	glycosylphosphatidylinositol
HA	hyaluronic acid
HIF	hypoxia inducible factor
HLA	human leukocyte antigen
HPV	human papilloma virus
HGF	hepatocyte growth factor
ICAM	intercellular adhesion molecule
IGF	insulin-like growth factor
JNK	jun N-terminus kinase
LPA	lysophosphatidic acid
LPS	lipopolysaccharide
MAPK	mitogen-activated protein kinase
MAT	micronized adipose tissue
MHC	major histocompatibility complex
MMP	matrix metalloproteinase
mRNA	messenger ribonucleic acid
miRNA	microribonucleic acid
MSC	mesenchymal stem cell
MCP-1	monocyte chemoattractant protein-1
NGF	nerve growth factor
Oct	octamer transcription factor
PCL	polycaprolactone
PDGFR	platelet-derived growth factor receptor
PEG	polyethylene glycol
PGA	polyglycolic acid
PI3K	phosphatidylinositol-3-kinase
PLA	polylactic acid
PLGA	poly(lactic-co-glycolic acid)
PMMA	polymethylmethacrylate
PODXL	podocalyxin
PRP	platelet-rich plasma
PU	polyurethane
ROS	reactive oxygen species
RNA	ribonucleic acid
SVF	stromal vascular fraction
TERT	telomerase reverse transcriptase
3D	three-dimensional
tPRP	thrombin-activated platelet-rich plasma
tRNA	transfer ribonucleic acid
VCAM	vascular cell adhesion molecule 1
VEGF	vascular endothelial growth factor
VLA-4	very late antigen-4
